# Pain thresholds and suprathreshold pain after sleep restriction in migraine – A blinded crossover study

**DOI:** 10.1177/03331024211056565

**Published:** 2021-11-17

**Authors:** Jan Petter Neverdahl, Martin Uglem, Dagfinn Matre, Johannes Orvin Hansen, Morten Engstrøm, Erling Tronvik, Lars Jacob Stovner, Trond Sand, Petter Moe Omland

**Affiliations:** 1 Department of Neuromedicine and Movement Sciences, Faculty of Medicine and Health Sciences, NTNU, Norwegian University of Science and Technology, Trondheim, Norway; 2 Department of Neurology and Clinical Neurophysiology, St. Olavs Hospital, Trondheim, Norway; 3 Norwegian National Headache Centre, St. Olavs Hospital, Trondheim, Norway; 4 Department of Work Psychology and Physiology, National Institute of Occupational Health, Oslo, Norway

**Keywords:** Pressure pain threshold, heat pain threshold, headache, allodynia, photophobia

## Abstract

**Objective:**

There is an unexplained association between disturbed sleep and migraine. In this blinded crossover study, we investigate if experimental sleep restriction has a different effect on pain thresholds and suprathreshold pain in interictal migraineurs and controls.

**Methods:**

Forearm heat pain thresholds and tolerance thresholds, and trapezius pressure pain thresholds and suprathreshold pain were measured in 39 interictal migraineurs and 31 healthy controls after two consecutive nights of partial sleep restriction and after habitual sleep.

**Results:**

The effect of sleep restriction was not significantly different between interictal migraineurs and controls in the primary analyses. Pressure pain thresholds tended to be lower (i.e., increased pain sensitivity) after sleep restriction in interictal migraineurs compared to controls with a 48-hour preictal-interictal cut-off (p = 0.061). We found decreased pain thresholds after sleep restriction in two of seven migraine subgroup comparisons: heat pain thresholds decreased in migraineurs with lower pain intensity during attacks (p = 0.005) and pressure pain thresholds decreased in migraineurs with higher severity of photophobia during attacks (p = 0.031). Heat pain thresholds tended to decrease after sleep restriction in sleep-related migraine (p = 0.060). Sleep restriction did not affect suprathreshold pain measurements in either group.

**Conclusion:**

This study could not provide strong evidence for an increased effect of sleep restriction on pain sensitivity in migraineurs compared to healthy controls. There might be a slightly increased effect of sleep restriction in migraineurs, detectable using large samples or more pronounced in certain migraine subgroups.

## Introduction

Sleep disturbances increase the risk of migraine attacks, and are frequently reported triggers (1), while adequate sleep seems protective (2). Migraineurs have poorer subjective sleep quality, more non-refreshing sleep, and might be relatively sleep deprived, i.e., having increased tiredness and slow-wave sleep despite normal sleep duration (3–5). Hence, there is a rather obvious but still unexplained relationship between sleep disturbances and migraine.

Migraineurs might experience allodynia during attacks, suggesting sensitisation of the trigeminovascular system (6,7). Presence of interictal allodynia has also been widely discussed. A recent meta-analysis found lowered heat pain threshold (HPT) and pressure pain threshold (PPT) in interictal migraineurs (8). For HPT, the pooled difference between migraineurs and controls was small, and a majority of studies found no differences (8). These results suggest that pain sensitisation generally is of small magnitude in interictal migraineurs. However, pain sensitisation may be more pronounced closer to the attacks and in clinical subgroups. For instance, migraineurs can be divided by sleep-related attack propensity or by sleep disturbance patterns (5).

Sleep restriction (SR) is a useful model for sleep disturbance in a pain context. A meta-analysis concluded that experimental SR increases pain perception with a medium effect in healthy subjects (9). Considering the relationship between sleep disturbance and migraine cited above, pain perception may be altered more by SR in migraine than in non-headache subjects. We hypothesised that SR induce more general sensitisation of pain pathways in migraineurs than in non-headache subjects, as measured by thermal and pressure pain threshold and suprathreshold tests. To our knowledge, no other study has investigated this hypothesis.

In the present blinded crossover study, the primary objective was to investigate the effect of SR on experimental pain responses in migraineurs. Therefore, the effect of SR on HPT, heat pain tolerance threshold (HPTT), PPT, and a suprathreshold pressure pain protocol were compared in interictal migraineurs and headache-free controls. Secondary objectives were: 1) to explore whether the effect of SR on experimental pain responses was different in subgroups of migraineurs, and if the effect of SR depends on clinical variables quantifying sleepiness, insomnia, and total sleep time, and 2) to reinvestigate, in a blinded study, pain perception differences between interictal migraineurs and healthy controls.

## Methods

### Design

**Figure 1.**
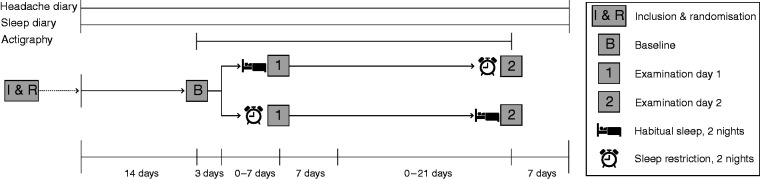
Overview of the study’s timeline. All participants completed sleep diary and wore an actigraph. Only migraineurs completed headache diaries. Participants slept for 4 hours for two consecutive nights (sleep restriction condition) prior to one of the examination days, and slept habitually prior to the other. The order of sleep conditions was balanced between examination days and randomised before commencement of each participant’s study period. Randomisation was done in blocks and separately for controls and migraineurs to ensure that the order of sleep conditions was similar in both groups throughout the study. The interval between Baseline and Day 1, and Day 1 and Day 2, could vary between 3-10 days and 1-4 weeks, respectively, to ensure sufficient flexibility for participants. The lower limit of 1 week was chosen to minimise potential residual sleep deprivation effects in the case of sleep restriction prior to Day 1.

**Figure 2.**
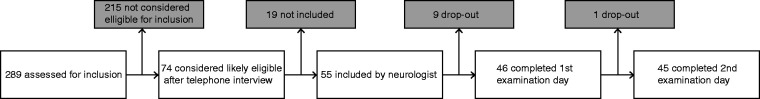
Flow chart showing inclusion of migraineurs in the study.

Participants visited our lab three times during the study: First for baseline testing (referred to as Baseline), and subsequently for two examination days (referred to as Day 1 and Day 2): Once after habitual sleep, and once after SR (Figure 1). During Baseline, the patients completed the procedure from subsequent examination days. The Baseline was added to familiarise the test subjects with the experimental procedures and to minimise learning effects between examination days. Order of sleep conditions was random and balanced between examination days. Participants met the same examiner each examination day. The examiner was blinded to diagnosis and sleep condition during data collection and data analysis. Participants were included in the winter and spring of 2016; data were collected from May to December in 2016 (Figure 2).

### Test subjects

Participants were recruited from the general population, by advertisement in our university. A study nurse screened migraineurs and controls in accordance with exclusion criteria (presented in Table 1) by telephone. Migraineurs were subsequently examined by a neurologist and diagnosed according to the ICHD-III (beta) criteria (10). Participants signed a written informed consent in accordance with the Declaration of Helsinki. The study was approved by the Regional Committee for Medical and Health Research Ethics.

**Table 1.** Exclusion criteria for all participants.

Co-existing tension type headache (≥7 attacks/month for migraineurs)
Neurological or psychiatric disorder with decreased function
Confirmed sleep disorder
Infectious disease
Connective tissue disorders
Metabolic, endocrine, or neuromuscular disease
Acute or chronic pain disease
Recent injury affecting function
Neoplastic disease
Previous craniotomy or cervical neurosurgery
Pregnancy
Cerebrovascular or symptomatic heart disease
Pulmonary disease
Hypertension (>160/110)
Pregnancy
Medication for acute or chronic pain
Neuroleptic or anti-epileptic drugs
Anti-depressive drugs
Cardiovascular, pulmonary, or antihypertensive drugs
Other drugs that might influence neuronal, vascular, or muscular function
Body mass index (BMI) <17 or >35
Alcohol or drug abuse
Ferromagnetic implants
Additional exclusion criteria for controls
≥1 minor headache per month
When occasional headaches, controls were not included if ≥1 of the following were affirmed
Consultation by a physician
The headache was experienced as painful
The headache caused use of abortive medication

Included episodic migraineurs had 2–6 migraine attacks/month, and ≤10 days with migraine/month. Forty-six migraineurs and 31 healthy, sex- and age-matched controls participated in our study. We did not compare migraineurs with and without aura, as pain perception seems similar between these subgroups (11) and the migraine with aura subgroup was small (Table 2).

**Table 2.** Demographic and clinical data after exclusions.

	Controls	Interictal migraineurs	
	≥1 recorded test day	≥1 interictal test day	2 interictal test days
Total number of subjects	31^a^	39^b^	21^c^
Age	36.2 (10.6)	39.2 (9.1)	35.5 (7.6)
Age range	20–56	20–60	20–48
BMI	24.4 (3.4)	24.3 (3.9)	23.8 (4.2)
Women/Men	23/8	32/7	15/6
MwoA/MwoA + MA/MA	NA	22/12/5	10/8/3
NSM/SM	NA	29/10	18/3
Days from last menstruation			
Before habitual sleep	29.3 (36.1)	29.4 (29.8)	32.1 (30.1)
Before sleep restriction	26.9 (38.3)	19.9 (20.3)	18.6 (22.4)
Use of hormonal contraception	9	16	10
Epworth sleepiness scale (0-24)	6.7 (4.0)	6.7 (3.8)	7.2 (4.1)
Insomnia symptom score (0-12)	3.6 (1.9)	4.8 (2.7)	4.9 (2.8)
Years with headache	NA	22.0 (10.2)	19.7 (10.9)
Migraine days/month^d^	NA	4.8 (2.9)	4.5 (2.8)
Migraine intensity (1–4)^e^	NA	2.8 (0.6)	2.8 (0.6)
Headache duration in hours^f^	NA	10.0 (14.7)	11.3 (17.3)

Data displayed as mean (SD) in selected cases. MwoA: migraine without aura. MA + MwoA: both attacks with and without aura (both diagnoses according to ICHD-III (beta) criteria). MA: migraine with aura (in 100% of attacks). NSM: non-sleep-related migraine (headache start “during daytime before noon”, “during daytime after noon”, or “no regular onset time”). SM: sleep related migraine (headache start “upon waking” or “during the night (waking me up)”). Demographic and variables from controls, the migraine population that had at least one interictal test day, and the subgroup of migraineurs that had two interictal test days. ^a^29 with both habitual sleep and SR in thermal analyses and in pressure analyses. ^b^36 in primary thermal analyses after exclusions; 38 and 35 in secondary pressure and thermal analyses, respectively. ^c^20 migraineurs were interictal both test days in primary thermal analyses; 16 and 15 in secondary pressure and thermal analyses, respectively. ^d^Days with migraine the last 3 months. ^e^Migraine intensity during attacks: 1: mild, 2: moderate, 3: severe, 4: extreme. ^f^Average duration of headache with or without use of medication. NA: Not applicable.

**Table 3.** Mean (SD) of selected sleep variables by diagnosis and sleep condition.

	Controls (N = 31)		Interictal migraineurs (N = 39)	
	Habitual sleep	Sleep restriction	Habitual sleep	Sleep restriction
n	31	31	30	30
Total sleep time (hours)^a^	7.0 (0.6)	3.9 (0.3)	6.7 (1.2)	3.7 (0.9)
Time in bed (hours)^b^	7.8 (1.0)	5.3 (1.4)	7.0 (1.3)	5.4 (1.3)
Karolinska sleepiness scale (1–9)^c^	2.2 (1.8)	5.5 (3.2)	3.0 (2.3)	6.3 (2.9)
Psychomotor vigilance test (1/s)^d^	3.1 (0.2)	3.1 (0.2)	3.1 (0.3)	3.0 (0.3)

N = number of test subjects that had at least one test day, either one after habitual sleep or SR, or both. N = number of test days after each sleep condition. ^a^Total sleep time was calculated based on actigraphy measurements (Cole-Kripke algorithm) from the two nights preceding each test day. The rest intervals defined by the actigraphy software were adjusted with lights off (intention to sleep) and out of bed time from sleep diary and light and activity levels from actigraph. ^b^Time in bed was calculated based on sleep diary. ^c^Karolinska sleepiness scale (1–9), measured after completion of testing each test day. ^d^Results from the psychomotor vigilance test (PVT, simple reaction time) were inverted (1/s), and the 10% smallest and largest values were removed.

Two migraineurs were excluded from all analyses because of withdrawal of consent, or failure to complete headache diary. Data from one examination day after sleep restriction from one migraineur was not used for further analysis because of incomplete headache diary pertaining to the relevant examination day. Data from both examination days from three migraineurs, one examination day after habitual sleep from one migraineur, and one examination day from two controls (one after habitual sleep; one after sleep restriction) were not used for further analysis for thermal thresholds because of a malfunction of the thermal stimulator. Pressure thresholds from one examination day in one migraineur after habitual sleep were not used in further analysis due to malfunction of the algometer. Pressure thresholds from one examination day in one control after habitual sleep were not used in further analysis due to failure to reach PPT before the safety limit of N = 100 was met.

These exclusions and decisions regarding use of data for further analysis were made prior to data analysis, and without knowledge of participant diagnosis or sleep condition. Number of participants after exclusions can be found in Table 2 and Supplementary Table S1. The measurements in migraineurs were classified by headache diaries as interictal if there was no migraine headache the day before or after examination. We used a 24-hour cut-off in accordance with previous studies on pain physiology and migraine phase (12), because premonitory phase symptoms reliably predict the attack within 24 hours (13). We also used a 48-hour cut-off in a secondary set of analyses, in line with recent recommendations (14).

### Procedure

Participants arrived at the same time both examination days, either 08.00 or 09.30, and were instructed to avoid use of nicotine or caffeine after midnight before examination days. Each examination day, in the following order, participants underwent: 1) structured interview assessing use of caffeine, alcohol, and nicotine in the 24 hours prior to examination days, current use of hormonal contraception, and time of last menstruation; 2) self-report of medication use, presence and character of any potential headache (the researcher was blinded to information in this form); 3) blood pressure measurement; 4) psychomotor vigilance test (PVT), a simple reaction time test lasting 10 minutes (custom-written C++ program, National Institute of Occupational Health, Norway). PVT was added to yield a quantifiable measure of alertness as a correlate to sleep deprivation; 5) five consecutive measurements of warm detection threshold (WDT); 6) followed by three heat pain threshold (HPT) measurements; 7) three heat pain tolerance threshold (HPTT) measurements; and 8) pressure thresholds once for each of the left and right trapezius muscles. This procedure was the first part of a larger neurophysiological data collection lasting approximately 2 hours, which also included measurements of conditioned pain modulation and somatosensory evoked potentials. At the end of the subsequent data collection, patients reported potential headache, and sleepiness measured by Karolinska sleepiness scale (KSS). The researcher was blinded to this information. Results from the PVT were inverted (1/s), and the 10% smallest and largest values were removed (15).

### Thermal stimuli

Heat stimuli were applied to the volar left arm, 2 cm proximal to the flexor groove, using a longitudinally placed hand-held rectangular 25 × 50 mm Peltier element thermode (Somedic Sales AB, Stockholm, Sweden). We used the method of limits; the temperature increasing 1°C/s from a baseline-temperature of 32°C, with a limit at 52°C. Participants pressed a button when the thermode felt warm for warm detection threshold (WDT), when pain was felt for HPT, and for worst imaginable pain for HPTT. For HPTT assessment, the thermode was removed when the button was pressed or the 52°C limit was reached. WDT was measured five times, HPT and HPTT three times. Interstimulus intervals varied randomly between 4-6 seconds.

### Pressure stimuli

Pressure stimuli were applied to the trapezius muscles, as a pilot study conducted by a collaborating group showed better repeatability for this location. The stimuli were applied at points 1/3 from the posterior edge of the acromion to the C7 (16), using a FDMIX digital hand-held force gauge instrument (Wagner instruments, Greenwich, USA, probe size 1 cm^2^. Using a 1 cm^2^ probe, measured force = 10 N correspond to a pressure = 100 kPa). We used a custom-written program (National Institute of Occupational Health, Norway) providing real-time visual feedback of force to the investigator, to ensure an increment of 50 kPa/sec (16). Pain levels were measured continuously by the patient using a hand-operated visual analogue scale device (VAS, 0–100 mm, National Institute of Occupational Health, Norway) with endpoints “no pain” and “worst imaginable pain”; the VAS measurements were recorded digitally from the VAS device. The applied force corresponding to VAS = 50 mm was reached and recorded as “suprathreshold pressure pain” (PP5); the first sensation of pain was reported by first movement of an indicator button on the VAS device (and the corresponding force was recorded as PPT). Hence, PPT and PP5 were measured in the same sequence. This sequence was conducted once for the left trapezius and once for the right trapezius muscle.

### Sleep restriction (SR)

A self-administered partial SR protocol was applied. When completing the SR condition, the participants were instructed to sleep 4 hours for two consecutive nights, corresponding to approximately 50% SR.

### Sleep and headache diaries

Participants completed sleep diaries (see Figure 1 and Supplementary Figure S3 for details) in the period from two weeks prior to Baseline, to one week after Day 2. Migraineurs completed headache diaries in parallel with sleep diaries. The headache diary included detailed written instructions, and migraineurs were instructed in using the headache diary by a study nurse. Details about the headache diary can be found in the Supplementary material. Both diaries have been used in previous studies by our group (17).

### Collection of clinical migraine and sleep variables

Total sleep time was recorded by wrist actigraph (Actiwatch Spectrum Plus, Philips Respironics, USA), and averaged over the two days preceding examination days. Rest intervals defined by the actigraphy software (Philips Actiware 6, Philips Respironics, USA) were corrected semi-manually (18). Daytime sleepiness was recorded with Karolinska sleepiness scale (KSS, score 1–9), once at the end of each examination day (approximately 2 hours after start) (19). Clinical sleep variables were collected by self-report form at home: Tendency to fall asleep at daytime was recorded by Epworth sleepiness scale (ESS, score 0–3 for eight questions, summed to a total of 24 (20)); insomnia symptoms were recorded with Insomnia symptom score (ISS, score 0–3 for four questions, summed to a total of 12) (21). Variables on clinical migraine traits were collected by structured interview by a research nurse: Data on intensity, frequency, and duration of headache, intensity and frequency of photophobia and phonophobia, time of diagnosis, and relation of headaches to sleep were used in analyses. Migraine headache was classified as sleep-related migraine (SM) if headaches usually started “upon waking” or “during the night (waking me up)”, and as non-sleep-related migraine (NSM) if headaches usually started “during daytime before noon”, “during daytime after noon”, or “no regular onset time” (17). Days from examination day to next attack was recorded by headache diary.

### Data analysis and statistical methods

#### Primary analyses

Thermal thresholds were defined as difference in °C from 32°C. Values of ≥3 times or ≤1/3 of the mean of the associated responses were excluded (12) (a total of 10 WDT-measurements were removed from five migraineurs and three controls (six after habitual sleep; two after SR) because these technical inclusion criteria were not met). PP5 was calculated by a linear fit between force and VAS ratings (Supplementary Figure S1) (16). A lack of linearity, defined as R^2^ < 0.80, resulted in exclusion of PP5 (a total of 4 PP5-measurements from two controls and two migraineurs (two after habitual sleep; two after SR) were removed because this technical inclusion criterion was not met).

We used STATA version 16.1 (StataCorp LLC) to run separate multilevel models for the primary (HPT, HPTT, PPT, and PP5) and secondary (WDT) response variables. The models included simple effects of “group” (migraine vs. controls) and “sleep” (SR vs. habitual sleep), and their interaction. The interaction (main outcome) determined whether the effect of SR compared with habitual sleep differed between interictal migraineurs and controls. The models were specified as two-level models with recordings nested in subjects and random slope for examination day. We specified a random intercept for subjects, a random coefficient for sleep for WDT, HPT and HPTT, and an unstructured variance-covariance matrix for HPT. Random parameters and covariance matrices were included based on likelihood ratio tests.

Normality of level one residuals and higher-level random effects was checked visually by histograms and qq-plots, and response variables were transformed when determined necessary. Maximum-likelihood estimation with sandwich estimator was used for WDT, HPT, and HPTT to account for less than normally distributed residuals, and restricted maximum-likelihood estimation for PPT and PP5 in the primary analysis. HPTT measurements ≥52°C were defined as censored in an additional Tobit regression analysis (22). See Appendix for full model specifications.

#### Secondary analysis

In a secondary analysis we repeated analyses of the primary and secondary response variables using a 48-hour cut-off for the interictal-preictal border (14). Number of participants for these analyses are shown in Supplementary Table S1.

#### Sample size and power calculation

In our experience, 45 migraineurs and 30 controls in a cross-sectional design yields approximately equal groups after excluding non-interictal migraineurs. 30 migraineurs and 30 healthy controls in a two-sample t-test generates approximately 70% power to detect a low medium-sized effect (0.65 SD). 20 migraineurs with two interictal examination days yields a power of 79% to detect a similar effect size (0.65 SD) in a paired t-test. Results producing p < 0.05 were considered significant. Multilevel models are well-equipped at handling missing data (23). Hence, in the cases where one examination day was excluded, the other examination day was still included in the analyses.

#### Exploratory analysis of the association between SR-effects on pain thresholds and suprathreshold pain, and clinical migraine and sleep variables

In the exploratory analyses, we extended the main models on HPT, HPTT, and PPT with addition of KSS, ESS, and ISS as covariates. In addition, we ran separate multilevel models in the migraine group only with main effects of “sleep” and a single clinical variable, and their interaction. We added post hoc contrasts to test simple effects in the cases of significant main effects or interactions. The clinical variables used were: 1) headache intensity during attacks, 2) duration of migraine headaches, 3) frequency of attacks, 4) years since diagnosis (age as covariate), 5) time to next attack, 6) intensity and frequency of photophobia and phonophobia, respectively, and 7) sleep-related migraine. Lastly, one model including main effects of “total sleep time” and “group”, and their interaction on HPT, HPTT, and PPT was run.

## Results

Demographic and clinical data are shown in Table 2. Based on mean values, participants in both groups slept close to the goal of 4 hours during SR nights, and sleep time in the SR condition was approximately 55% SR relative to the habitual sleep condition on the group level for both controls and migraineurs (Table 3). 5 test subjects had ≤2 hours difference in mean total sleep time between the habitual sleep and SR conditions (4 migraineurs; 1 control).

### Interactions between group factor (migraine vs. control) and sleep factor (SR vs. habitual sleep)

**Figure 3.**
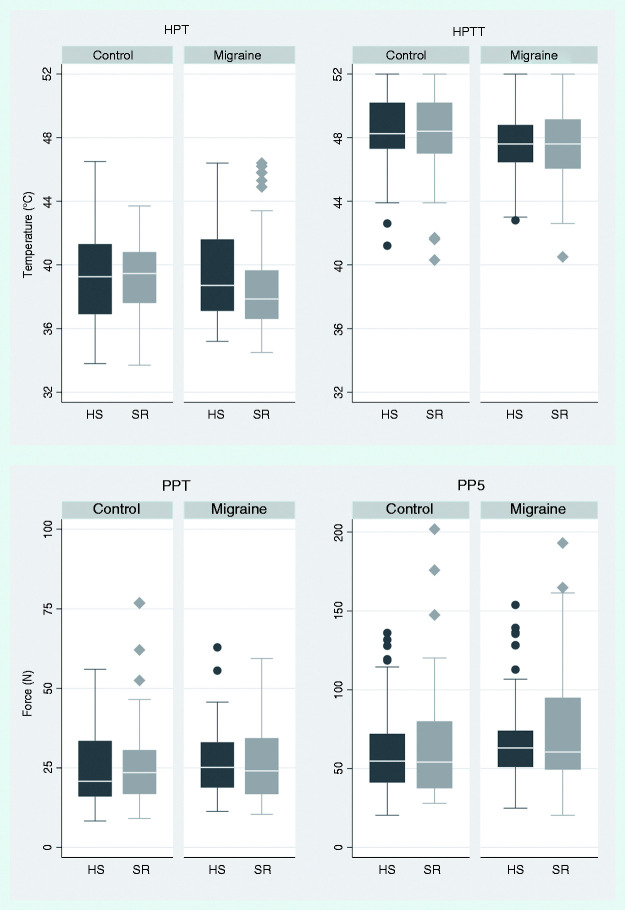
Box plot of the primary response variables: heat pain threshold (HPT) and heat pain tolerance threshold (HPTT) (above), pressure pain threshold (PPT) and pressure pain at VAS = 50/100 (PP5) (below) for each sleep condition and group. The box plot shows the 25th and 75th percentile as borders, and the median as a line. The whiskers show upper and lower adjacent values. Circles and diamonds show outliers. PP5 measurements from one test subject are omitted from this figure, as they were impractically large for depiction (HS: 256 N, SR: 415 N). Residual diagnostics were acceptable, and transformed values were normally distributed, so these values were not excluded from analyses. Using a 1 cm^2^ probe, 10 N correspond to 100 kPa. Note that thermal thresholds use the same range for the y-axis, while pressure thresholds do not.

**Figure 4.**
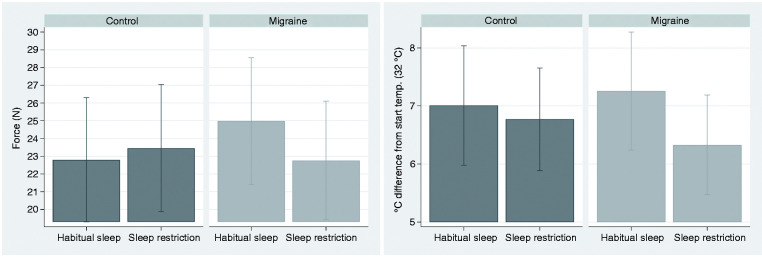
Pressure pain threshold (PPT) (left) and heat pain threshold (HPT) (right) in a secondary analysis using a 48-hour cut-off for the preictal phase. Graphical display of estimated margins with 95% confidence intervals from a multilevel model, showing the effect of habitual versus restricted sleep (SR) in interictal migraineurs and controls. PPT tended to be lowered more by SR in migraineurs than in controls. For HPT, the point estimate was lower after SR in migraineurs, but the finding was not significant. P-values for all effects are shown in Table S2.

SR did not have a significantly different effect on pain thresholds (HPT, PPT) and suprathreshold pain measurements (HPTT, PP5) in migraineurs compared to controls (p > 0.15, Table 4, Figure 3). SR tended to decrease PPT (i.e., increase pain sensitivity) relatively more in migraineurs compared to controls (p = 0.061, Supplementary Table S2, Figure 4) in the secondary analysis using a 48-hour cut-off (see section on test subjects, last paragraph). This trend relates to higher pressure pain sensitivity after SR compared with habitual sleep in the migraine group (p = 0.070). The point estimate for HPT was lower after SR in the migraine group in the secondary analysis, but not significantly lower (p = 0.10).

**Table 4.** Thermal and pressure thresholds in interictal migraineurs and controls after habitual sleep and restricted sleep for the primary response variables.

		Heat pain threshold (HPT)			Heat pain tolerance threshold (HPTT)		
		Difference from 32 °C [95% CI]			Difference from 32 °C [95% CI]		
	n	Habitual sleep	Sleep restriction	p-value	Habitual sleep	Sleep restriction	p-value
Control	31	7.0 [6.0, 8.0]	6.8 [5.9, 7.6]	p = 0.55^a^	16.7 [16.0, 17.5]	16.6 [15.8, 17.4]	p = 0.49^a^
Migraine	36	7.2 [6.2, 8.1]	6.4 [5.5, 7.3]	p = 0.11^b^	15.7 [15.0, 16.4]	15.5 [14.7, 16.3]	p = 0.62^b^
p-value		p = 0.82^c^	p = 0.56^d^	p = 0.40^e^	p = 0.051^c^	p = 0.063^d^	p = 0.96^e^

		Pressure pain threshold (PPT)			Pressure at VAS = 50/100 (PP5)		
		Force (N) [95% CI]			Force (N) [95% CI]		
Control	30	22.8 [19.3, 26.3]	23.5 [19.9, 27.0]	p = 0.47^a^	54.1 [45.6, 62.7]	54.9 [46.2, 63.6]	p = 0.66^a^
Migraine	39	25.0 [21.5, 28.4]	23.6 [20.3, 26.9]	p = 0.21^b^	64.6 [55.0, 74.2]	65.9 [56.0, 75.7]	p = 0.62^b^
p-value		p = 0.39^c^	p = 0.95^d^	p = 0.15^e^	p = 0.11^c^	p = 0.102^d^	p = 0.93^e^

CI: confidence interval. N: = number of test subjects that had at least one test day, either one after habitual sleep, one after sleep restriction (SR), or two, i.e., one after each sleep condition. (N): Newton. Predicted means with 95% confidence intervals from the primary analysis. Means are shown as difference from baseline temperature of 32°C, and absolute force (using a 1 cm^2^ probe, 10 N correspond to 100 kPa). PP5 was calculated based on a linear fit between force and pain (Supplementary Figure S1). ^a,b^P-values of the difference between the two sleep conditions in the control and migraine groups, respectively. ^c,d^P-values of the difference between migraineurs and controls for habitual sleep and SR, respectively. ^e^P-value of the interaction between group and sleep condition. Random parameters are shown in Supplementary Table S4.

The secondary response variable WDT produced no significant results (Table 5).

**Table 5.** Warm detection threshold (WDT, secondary response variable) in interictal migraineurs and controls after habitual sleep and restricted sleep.

		Warm detection threshold (WDT)		
		Difference from 32°C [95% CI]		
	n	Habitual sleep	Sleep restriction	p-value
Control	31	1.5 [1.3, 1.6]	1.5 [1.3, 1.7]	p = 0.47^a^
Migraine	36	1.7 [1.4, 1.9]	1.7 [1.4, 2.0]	p = 0.66^b^
p-value		p = 0.19^c^	p = 0.24^d^	p = 0.88^e^

CI: confidence interval. N: = number of test subjects that had at least one test day, either one after habitual sleep, one after sleep restriction (SR), or one after both sleep conditions. Predicted means with 95% confidence interval from the primary analysis. ^a,b^P-values of the difference between the two sleep conditions in the control and migraine groups, respectively. ^c,d^P-values of the difference between migraineurs and controls for habitual sleep and SR, respectively. ^e^P-value of the interaction between group and sleep condition. Random parameters are shown in Supplementary Table S6.

### Simple effects of group (migraineurs vs. healthy controls)

**Figure 5.**
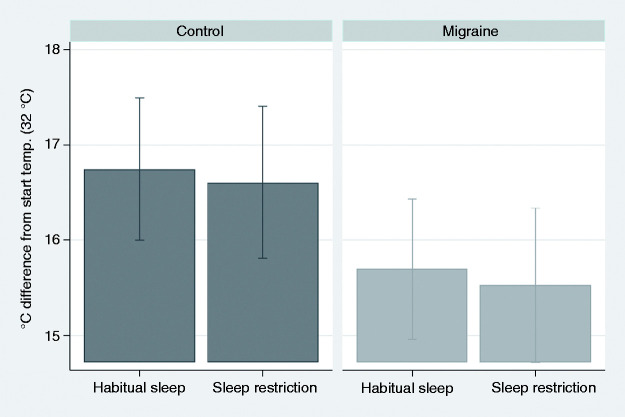
Heat pain tolerance threshold (HPTT) in the primary analysis, using a 24-hour cut-off for the preictal phase. Graphical display of estimated margins with 95% confidence intervals from the main multilevel model, showing the effects of habitual and restricted sleep in interictal migraineurs and controls. HPTT tended to be lower in migraineurs for both sleep conditions. P-values for the simple effects are shown in Table 4.

Thermal and pressure pain thresholds were not significantly different between migraineurs and controls in the primary analysis for the habitual sleep condition. HPTT tended to be decreased (i.e., increased suprathreshold pain sensitivity) in migraineurs compared to controls for both sleep conditions (p = 0.051 and p = 0.063, Table 4, Figure 5). In the secondary analysis (48-hour preictal cut-off), HPTT was decreased in migraineurs compared to controls after SR (p = 0.043, Supplementary Table S2). The Tobit regression analysis defining values ≥52°C as censored produced similar results.

### Migraine subgroups and clinical variables

**Figure 6.**
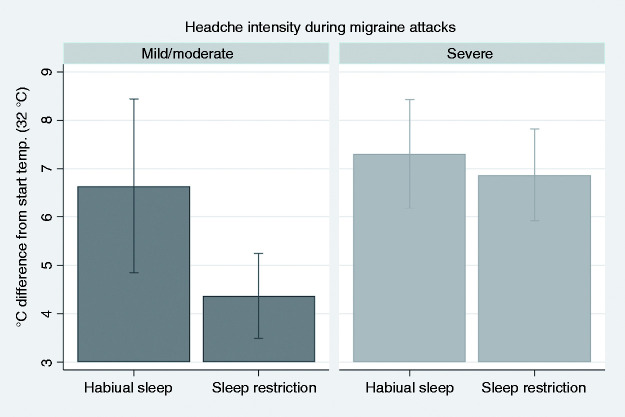
Heat pain threshold (HPT). Graphical display of estimated margins with 95% confidence intervals from a multilevel model, showing the effect of habitual vs. restricted sleep (SR) in migraineurs with mild/moderate compared with severe headache intensity during attacks. HPT was lower after SR compared to habitual sleep in the mild/moderate group. HPT was different between the groups for the SR condition.

The interaction between headache intensity group and sleep condition was significant (p = 0.045, Figure 6). Post hoc analysis of contrasts showed a significant difference between the intensity groups after SR (p < 0.001), and of SR in the mild/moderate group (p = 0.005); i.e., SR has a greater effect on HPT in migraineurs with mild/moderate headache intensity during attacks. There was an analogous trend toward lower HPTT in the mild/moderate intensity group compared to the severe headache intensity group (p = 0.058, Supplementary Table S8).

**Figure 7.**
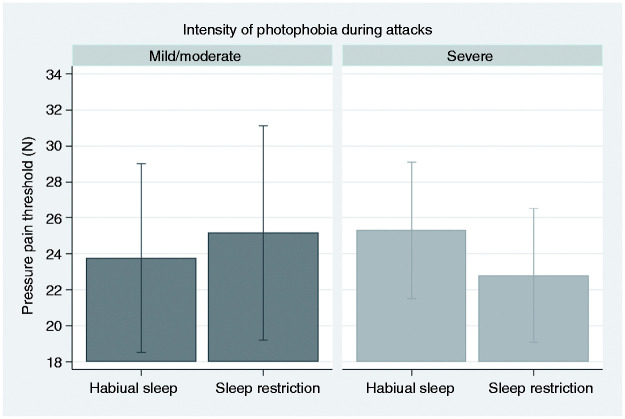
Pressure pain threshold (PPT). Graphical display of estimated margins with 95% confidence intervals from a multilevel model, showing the effect of habitual versus restricted sleep (SR) in migraineurs with mild/moderate compared with severe intensity of photophobia during migraine attacks. PPT was lower after SR in the group with severe photophobia, with a significant interaction between photophobia group and sleep condition. Using a 1 cm^2^ probe, 10 N correspond to 100 kPa.

For PPT, there was a significant interaction between photophobia intensity group and sleep condition (p = 0.019, Figure 7). Post hoc analysis of contrasts showed that SR decreased PPT only in the group with severe photophobia (p = 0.031).

HPT was decreased after SR in migraineurs in the model including NSM and SM patients (p = 0.040, Supplementary Figure S2). Post hoc analysis of contrast showed that this main effect stemmed mainly from a trend in the SM group (p = 0.060). HPT tended to be lower in the NSM group for the habitual sleep condition (p = 0.071).

HPTT increased with increasing duration of migraine attacks (p = 0.049). We could not find significant effects of KSS, ESS, ISS, or total sleep time in models including interictal migraineurs and healthy controls (p > 0.19), or of frequency of attacks, years with diagnosis, frequency of photo- or phonophobia, intensity of phonophobia, or time to next attack in models including only interictal migraineurs (p > 0.13).

## Discussion

We found no effect of SR on pain thresholds (HPT and PPT) or suprathreshold pain measurements (HPTT and PP5) in healthy controls. The effect of SR was not different in migraineurs and controls in the primary analyses. However, PPT tended to decrease more after SR in interictal migraineurs than in controls using the stricter 48-hour cut-off for the interictal-preictal border. Although an effect of SR was not present in the majority of explorative analyses, SR decreased HPT in interictal migraineurs with mild/moderate headache and PPT in interictal migraineurs with severe photophobia; similarly, SR tended to decrease HPT more in a subgroup with sleep-related migraine than in non-sleep-related migraine.

We found no statistically significant differences in HPT or PPT between interictal migraineurs and healthy controls in the primary analyses, although there were trends toward lower HPTT in migraineurs for both sleep conditions. In the secondary analysis this difference was significant for the SR condition.

### The effect of SR on pain measures in migraineurs

To our knowledge this is the first study investigating experimental pain perception in migraineurs after SR. Our findings of increased non-cephalic trapezius pressure pain sensitivity (lower PPT) in the secondary analysis suggest that SR may induce generalised sensitisation in migraineurs to a higher degree than in healthy controls (7). Similarly, HPT was lower after SR in migraineurs compared to controls, although not significantly, which could be due to the large between-subject variation (Figure 3, upper left panel and Figure 4, right panel).

HPT and PPT were similar in interictal migraineurs and controls after habitual sleep, and before and after SR in healthy controls. Meta-analyses have found increased pain sensitivity in interictal migraineurs compared to healthy controls (8), and after SR compared to HS, in healthy controls (9). Although several other studies have not found these differences, no published study report *reduced* pain sensitivity in interictal migraineurs compared to controls, nor in healthy subjects after SR (8,9). Hence, our findings support a subclinical state of hypersensitivity in migraineurs, manifesting as a slightly decreased group-mean pain threshold in the presence of a trigger such as SR (7). However, reliable detection of this small effect might require large sample sizes.

The neurophysiological correlates of SR are not well characterized. In healthy controls, SR might alter cortical inhibitory function (24), as well as serotonergic (25), opioidergic (26), and dopaminergic (27) neurotransmission. Some studies have found reduced conditioned pain modulation (CPM), a measure of endogenous pain modulation (28), after SR in healthy controls (29,30). Inhibitory cortical function might also be altered in migraineurs (31), possibly due to thalamocortical dysrhythmia and thalamic disconnection from brain stem serotonergic pathways (32,33). Similarly, migraineurs might have reduced CPM (34). One study found normal CPM in migraineurs using a standard protocol, but diminishing CPM after repeated testing (35). Thus, the possible hypersensitivity in migraineurs might be due to subtle alterations in endogenous pain modulation in migraineurs, that might be altered further by SR.

Some migraineurs do not experience allodynia during attacks. This group of migraineurs might not be susceptible to local or widespread pain sensitisation for instance after SR (6). Such neurophysiological differences might follow clinical traits (32) and explain why the explorative analysis indicated that SR decreased pain thresholds in one of the migraine intensity subgroups and one of the photophobia intensity subgroups. Ictal photophobia and allodynia tend to co-exist (36). Dura-sensitive neurons in the posterior thalamus receive input from photosensitive retinal cells, offering a link for this relationship (37). It is unclear why SR seems to affect HPT in migraineurs with mild-to-moderate and not severe headache intensity. Participants were not asked about allodynia during attacks for this study, and therefore it is not known if the group with mild-to-moderate headache intensity also had a higher degree of allodynia during attacks.

Increased heat pain sensitivity has been found in non-sleep-related migraine (NSM), but not in sleep-related migraine (SM) patients (17). Similarly, we found trends toward increased heat pain sensitivity (lower HPT) in NSM compared to SM patients, and after SR in the SM group. NSM, but not SM, patients may be relatively sleep-deprived (17). Hence, NSM patients may be sensitised by SR at baseline, explaining both the higher pain sensitivity, and the low SR response in the NSM-group; possibly due to a physiological ceiling-effect.

We could not find significant effects of SR on pain thresholds in migraineurs using a 24-hour cut-off. However, we found trends toward increased pressure pain sensitivity (lower PPT) in migraineurs in the 48-hour analysis. Interestingly, Uglem et al. (12) found preictal hypoalgesia and, when excluding preictal measurements, increased pain sensitivity closer to the next attack. Hence, pain sensitivity may actually be decreased in the early preictal phase, before increasing a few hours before the attack (7,12). A 48-hour cut-off may accordingly result in a ‘cleaner’ interictal phase group, possibly explaining why this cut-off produced slightly larger pain-sensitisation effects in our study. 72-hour cut-offs have also been used to show preictal changes in migraine for VEP (38) and BAEP (39).

### Pain thresholds in interictal migraineurs

We found normal forearm HPT and trapezius PPT in interictal migraineurs after habitual sleep, in contrast to a meta-analysis showing lowered HPT generally, and lowered PPT in an aggregated head and neck group (8). Our pressure measurements are primarily non-cephalic from an innervation standpoint (C2-C4), although some trapezius nociceptive information involves the trigeminocervical complex (trigeminal nucleus caudalis, C1, and C2) and the accessory nerve (40). For HPT, the pooled difference between migraineurs and controls in a meta-analysis was small, a majority of the reported studies found no differences, while only a minority reported use of blinding (8). In contrast to PPT, stimulus location did not seem to affect HPT in Nahman-Averbuch et al. (8). There is no obvious pattern in the differing methodology, as reasonably well-powered, blinded studies measuring both in trigeminal and peripheral areas have found both unaltered (12), and reduced HPT (5,17) in interictal migraineurs.

### Suprathreshold pain in interictal migraineurs

Pain tolerance is a complex and multifactorial construct, unrelated to ageing (41) and affected by psychological factors (42). Suprathreshold pain measurements are particularly useful with tonic stimulation protocols, since temporal summation of pain, a sign of possible central sensitisation, can be detected (43), but this was not included in the present study. Decreased pressure pain tolerance threshold and PPT was interpreted as spreading central sensitisation in lateral epicondylitis (44) and low pressure pain tolerance threshold may predict treatment responses in fibromyalgia (45). Point-estimations of both threshold and suprathreshold pain quantifies the stimulus-response curve and helps to identify hyperalgesia, although no recommended method seems to exist (46). Accordingly, suprathreshold pain thresholds seem to reflect multifactorial aspects of mainly central pain perception.

Suprathreshold pain have mostly been reported as normal in migraine (8), but previous studies are small: Two studies found normal suprathreshold pressure pain in migraineurs (8). One study found decreased HPTT in migraineurs, comparable to our findings (47). However, a meta-analysis combining suprathreshold heat pain (laser and thermode) showed no differences between migraineurs and controls (8). In the present blinded study, we extend previous results from the literature, as we observed significantly higher suprathreshold heat pain sensitivity (decreased HPTT) in migraineurs compared to controls in the secondary analysis, and trends in the primary analysis. These findings add some support to the general hypothesis, that pain perception between attacks in migraineurs is subtly altered, depending on pain modality, location, and pain intensity (8).

### Strengths and limitations

Strengths of the present study include use of blinding, a rather large sample size, and a paired crossover design. Blinding procedures reduce risk of biased results (48), and their importance have received some attention recently (49). The SM subgroup was small (n = 9) and the results for this subgroup should be interpreted with caution.

One limitation is measurements from only non-cephalic locations. However, our aim was restricted to study the hypothesised generalised pain sensitisation in migraine. We did not explore possible sex-related differences because we expected to recruit few males. It might be considered if total sleep deprivation would cause larger effects. However, we chose two consecutive nights of partial, not total, SR, because partial SR is more tolerable and a more realistic model for insomnia and/or short sleep, and because both partial SR (50), and total sleep deprivation (29), have been shown to increase pain sensitivity in healthy subjects. The HPTs measured in this study were low compared to other reported means and reference values, although other data materials also seem to have a portion of relatively low HPT with lower limits extending below 37 °C (51,52). Despite the mean difference, the 5% confidence range among our controls (35.5 °C), was identical to the young female group in Magerl et al. (52) (35.6 °C). We measured detection, pain, and tolerance thresholds on Baseline as well as the two examination days, that should help participants understand the differences between detection, pain, and tolerance. Indeed, PPTs were more in line with reference values (53). The reason for the relatively low mean HPT is unclear, but stimulation location (forearm) and a young and predominantly female study population may have contributed (52).

SR might trigger migraine attacks (1), leading to more preictal migraineurs after this sleep condition. Preictal symptoms might start 72 hours prior to an attack but seem to be largely specific to the last 24 hours preceding the attack (13). We included only interictal measurements, and our findings in migraineurs after SR using a 48-hour cut-off should therefore predominantly reflect an effect of SR.

## Conclusion

In this blinded paired crossover study, we could not provide strong evidence for SR having a different effect on pain perception in migraineurs compared to controls. Pressure pain sensitivity tended to increase in migraineurs after SR using a stricter cut-off for the interictal-preictal border, while the effect of SR on heat pain thresholds did not reach significance, possibly due to large interindividual variability. Heat pain tolerance, tending to be lower in migraine compared to controls, was seemingly not affected by SR. Suprathreshold measurements likely reflect different aspects of pain than pain thresholds, but is less studied in migraine.

We conclude that two nights of restricted sleep does not appear to have large or clinically relevant effects on pain perception between attacks for all migraineurs. No published study has reported *reduced* pain sensitivity, neither in migraineurs compared to controls nor in healthy subjects after SR. The combined evidence from our and previous studies suggest that there is a probable true small effect of partial SR both in migraine and controls. This effect may be more pronounced in certain clinically defined migraine subgroups, possibly reflecting pathophysiological differences, for instance between sleep-related and non-sleep related migraineurs. Future research could also use extended or total SR and/or sleep-stage specific disruption protocols, record pain sensitivity both in cephalic and non-cephalic areas and include tonic suprathreshold measurements.

## Article highlights

- Partial sleep restriction had no effect on pain sensitivity in migraineurs or controls in the primary analysis in this study but tended to decrease pressure pain thresholds slightly more in migraineurs than in controls when using a stricter cut-off for the preictal-interictal border.
- Headache attack intensity, presence of photophobia, and attack onset during night-time seemed to modify the effect of sleep restriction on pain thresholds.

## Supplemental Material

sj-pdf-1-cep-10.1177_03331024211056565 - Supplemental material for Pain thresholds and suprathreshold pain after sleep restriction in migraine – A blinded crossover study Click here for additional data file. Supplemental material, sj-pdf-1-cep-10.1177_03331024211056565 for Pain thresholds and suprathreshold pain after sleep restriction in migraine – A blinded crossover study by Jan Petter Neverdahl, Martin Uglem, Dagfinn Matre, Johannes Orvin Hansen, Morten Engstrøm, Erling Tronvik, Lars Jacob Stovner, Trond Sand and Petter Moe Omland in Cephalalgia
