# Supplementary material for: Pain thresholds and suprathreshold pain after sleep restriction in migraine – A blinded crossover study
Source: Cephalalgia. 2021 Nov 17;42(6):466–80. doi: 10.1177/03331024211056565 (PMC9039317; doi:10.1177/03331024211056565)
Supplement: sj-pdf-1-cep-10.1177_03331024211056565 - Supplemental material for Pain thresholds and suprathreshold pain after sleep restriction in migraine – A blinded crossover study [file sj-pdf-1-cep-10.1177_03331024211056565.pdf]

Supplementary Table S1. Number of included test days for thermal (WDT, HPT, HPTT) and pressure analyses (PPT, PP5) in the primary and secondary analyses, using 24- and 48-hour cutoffs for the preictal phase, respectively.

	Thermal analyses				Pressure analyses			
	N		Habitual sleep	Sleep restriction	N		Habitual sleep	Sleep restriction
Controls	31	n	30	30	31	n	29	31
Migraineurs								
Primary analysis	36[a]	n	28	28	39[b]	n	30	30
Secondary analysis	35[c]	n	27	23	38[d]	n	29	25

WDT: warm detection threshold. HPT: heat pain threshold. HPTT: Heat pain tolerance threshold. PPT: Pressure pain threshold. PP5: pressure at VAS = 50/100. N = number of interictal test subjects that completed at least one test day; either one after habitual sleep, sleep restriction, or both. n = number of interictal test days after each sleep condition. [a]20 were interictal both test days (interictal pairs). [b]21 interictal pairs. [c]15 interictal pairs, [d]16 interictal pairs.

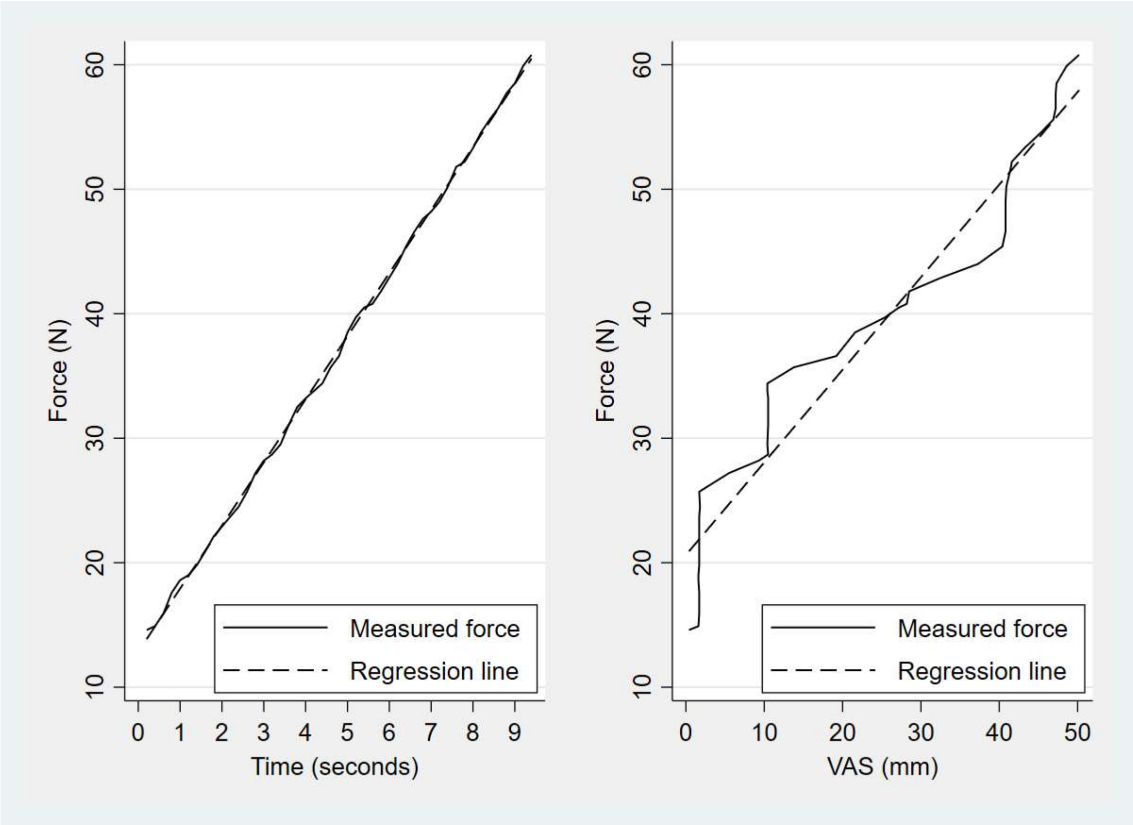

Figure S1. Example of pressure measurements using an algometer from the left trapezius of one test subject. Left: force in Newton (N) with time elapsed during one recording (using a 1 cm^2 probe, 10 N correspond to 100 kPa). The solid line is the actual force applied plotted against time elapsed, superimposed on a fitted line (dashed). Right: subjective pain on a visual analogue scale (VAS) in response to force. The solid line shows the VAS scored by the test subject on a manual VAS device as force against the trapezius muscle was increased with a constant rate. The dashed line is the fitted regression lines from measurements of force and VAS. These regression lines with their corresponding correlation coefficients are used to calculate pressure pain threshold (PPT) and pressure at VAS = 50/100 (PP5). The recordings were excluded in the case of $R^2 < 0.80$. In this case, R^2 was 0.95 for both the left and right sides. We used a FDMIX digital hand-held force gauge instrument (Wagner instruments, Greenwich, U.S.A., probe size 1 cm^2) to apply pressure to the trapezius muscles, at points 1/3 from the posterior edge of the acromion to the C7. A custom-written program (National Institute of Occupational Health, Norway) providing real-time visual feedback of force, was used to ensure an increment of 50 kPa/sec [16].

Supplementary Table S2. Pain and tolerance thresholds in interictal migraineurs and controls after habitual sleep and restricted sleep for the primary response variables in the secondary analysis, using a 48-hour cutoff for the preictal phase.

		Heat pain threshold (HPT)			Heat pain tolerance threshold (HPTT)		
		Difference from 32 °C [95% CI]			Difference from 32 °C [95% CI]		
	N	Habitual sleep	Sleep restriction	p-value	Habitual sleep	Sleep restriction	p-value
Control	31	7.0 [6.0, 8.0]	6.8 [5.9, 7.7]	p = 0.56[a]	16.7 [16.0, 17.5]	16.6 [15.8, 17.4]	p = 0.56[a]
Migraine	35	7.3 [6.2, 8.3]	6.3 [5.5, 7.2]	p = 0.102[b]	15.8 [15.0, 16.6]	15.3 [14.4, 16.2]	p = 0.18[b]
p-value		p = 0.74[c]	p = 0.48[d]	p = 0.32[e]	p = 0.071[c]	p = 0.043[d]	p = 0.51[e]

		Pressure pain threshold (PPT)			Pressure at VAS = 50/100 (PP5)		
		Force (N) [95% CI]			Force (N) [95% CI]		
	N	Habitual sleep	Sleep restriction	p-value	Habitual sleep	Sleep restriction	p-value
Control	31	22.8 [19.3, 26.3]	23.5 [19.9, 27.0]	p = 0.47[a]	54.1 [45.6, 62.6]	54.9 [46.3, 63.5]	p = 0.66[a]
Migraine	38	25.0 [21.4, 28.6]	22.8 [19.4, 26.1]	p = 0.070[b]	64.4 [54.7, 74.1]	63.5 [53.8, 74.1]	p = 0.74[b]
p-value		p = 0.39[c]	p = 0.78[d]	p = 0.061[e]	p = 0.12[c]	p = 0.20[d]	p = 0.60[e]

CI: confidence interval. N: = number of test subjects that had at least one test day, either one after habitual sleep, one after sleep restriction (SR), or two, i.e. after each sleep condition. (N): Newton. Predicted means with 95% CI from multilevel models. Means are shown as difference from baseline temperature of 32 °C, and absolute force (N). (using a 1cm^2 probe, 10 N correspond to 100 kPa). PP5 was calculated based on a linear fit for force plotted against pain from a visual analogue scale (VAS). [a,b]P-values of the difference between the two sleep conditions in the control and migraine groups, respectively. [c,d]P-values of the difference between migraineurs and controls for habitual sleep and restricted sleep, respectively. [e]P-value of the interaction between group and sleep condition. Random parameters are shown in Table S5. There was a significantly higher suprathreshold heat pain sensitivity in the migraineurs for the SR condition, and an analogous trend for the habitual sleep condition. Except for the fluctuations relative to the thresholds for significance, these findings are quite similar to those of the primary analysis. E.g. the difference in point estimates between migraine and control group for HPTT was 1.1 °C in the primary analysis, and 1.3 °C in the secondary analysis. In this analysis, there were trends toward higher heat and pressure pain sensitivity after SR (PPT and HPT), but only for the migraine subgroup. For pressure pain, there was also a trend toward a different effect of SR between the migraineurs and controls. Suprathreshold pressure pain sensitivity (PP5) seems to be lower in migraineurs based on the point estimates, but were not significant for neither sleep conditions, due to large variance.

Supplementary Table S3. Warm detection threshold (WDT, secondary response variable) in interictal migraineurs and controls after habitual sleep and restricted sleep for the secondary analysis, using a 48-hour cutoff for the preictal phase.

		Warm detection threshold (WDT)		
		Difference from 32 °C [95% CI]		
	n	Habitual sleep	Sleep restriction	p-value
Control	31	1.5 [1.3, 1.6]	1.5 [1.3, 1.7]	p = 0.48[a]
Migraine	35	1.7 [1.4, 1.9]	1.6 [1.3. 1.9]	p = 0.60[b]
p-value		p = 0.17[c]	p = 0.50[d]	p = 0.39[e]

CI: confidence interval. n: = number of test subjects that had at least one test day, either one after habitual sleep, one after sleep restriction (SR), or two, i.e. one after each sleep condition. Predicted means with 95% CI from multilevel models. Means are shown as difference from baseline temperature of 32 °C. [a, b]P-values of habitual sleep vs SR in controls and migraine, respectively. [d]P-value of controls vs migraine after habitual sleep and SR, respectively. [e]Represents the interaction between group and sleep condition. Random parameters are shown in Table S6. There were no significant findings.

Supplementary Table S4. Model specifications for the primary response variables in the primary analysis, using a 24-hour cutoff for the preictal phase.

	HPT (◦C$^{0.5}$)		HPTT (◦C^{2})	
	Coef.	[95 % CI]	Coef.	[95 % CI]
Simple effects				
Group	0.030	[-0.234, 0.294]	-33.97[a]	[-68.10, 0.154]
Sleep	-0.047	[-0.199, 0.106]	-4.619	[-17.80, 8.558]
Interaction				
Group x Sleep	-0.102	[-0.340, 0.136]	-0.707	[-25.47, 24.08]
Constant	2.647	[2.453, 2.842]	280.415	[255.4, 305.5]
Random effects		Estimate		Estimate
Level 2: subject (intercept)		0.256		4334.3
Level 2: session (slope)		0.162		1333.9
Level 1: residuals		-0.104		596.2
Covariance: (subject, session)		0.034		
	PPT (lnN)		PP5 (N$^{-0.3}$)	
	Coef.	[95 % CI]	Coef.	[95 % CI]
Simple effects				
Group	0.091	[-0.115, 0.297]	-0.016	[-0.035, 0.004]
Sleep	0.029	[-0.048, 0.105]	-0.001	[-0.007, 0.004]
Interaction				
Group x Sleep	-0.085	[-0.202, 0.032]	-0.000	[-0.009, 0.008]
Constant	3.127	[2.975, 3.278]	0.302	[0.288, 0.316]
Random effects		Estimate		Estimate
Level 2: subject (intercept)		0.160		0.001
Level 1: residuals		0.045		0.000

HPT: heat pain threshold. HPTT: heat pain tolerance threshold. CI: confidence interval. PPT: pressure pain threshold. PP5: pressure at VAS = 50/100. Ln: natural logarithm. N: Newton. HPT was square-root transformed, while HPTT and PP5 were power transformed, and PPT was log transformed, to improve normality of residuals. Coefficients presented are transformed and should be interpreted as such. The constant refers to mean thresholds for the control group for the habitual sleep condition. Simple effect of group: change in thresholds in migraineurs compared with controls for the habitual sleep condition. Simple effect of sleep: change in threshold after sleep restriction (SR) compared to habitual sleep for controls. The interaction refers to the difference between the effect of SR compared to habitual sleep in migraine compared to controls. Random effects are presented as variances. Random parameters and covariance matrices were included based on likelihood ratio (LR) tests. An unstructured variance-covariance matrix was used for HPT. We used maximum likelihood estimation (MLE) for HPT and HPTT, whereas restricted maximum-likelihood estimation (REML) was used for PPT and PP5. For HPT and HPTT we used the sandwich estimator, to account for less than normally distributed residuals. [a]p = 0.051.

Supplementary Table S5. Model specifications for the primary response variables in the secondary analysis, using a 48-hour cutoff for the preictal phase.

	HPT (◦$C^{0.5}$)		HPTT (◦C^2)	
	Coef.	[95 % CI]	Coef.	[95 % CI]
Simple effects				
Group	0.047	[-0.224, 0.317]	-31.39[a]	[-65.45, 2.672]
Sleep	-0.045	[-0.198, 0.108]	-4.609	[-20.05, 10.83]
Interaction				
Group × Sleep	-0.133	[-0.395, 0.130]	-9.464	[-35.05, 16.13]
Constant	2.647	[2.453, 2.842]	280.4	[256.1, 304.7]
Random effects		Estimate		Estimate
Level 2: subject (intercept)		0.264		4527.851
Level 2: session (slope)		0.173		1421.903
Level 1: residuals		0.034		587.741
Covariance: (subject, session)		-0.121		
	PPT (lnN)		PP5 ($N^{-0.3}$)	
	Coef.	[95 % CI]	Coef.	[95 % CI]
Simple effects				
Group	0.091	[-0.119, 0.301]	-0.015	[-0.035, 0.039]
Sleep	0.029	[-0.049, 0.106]	-0.001	[-0.007, 0.004]
Interaction				
Group × Sleep	-0.121[b]	[-0.248, 0.006]	0.003	[-0.007, 0.012]
Constant	3.127	[2.973, 3.280]	0.302	[0.287, 0.316]
Random effects		Estimate		Estimate
Level 2: subject (intercept)		0.165		0.001
Level 1: residuals		0.046		0.000

HPT: heat pain threshold. HPTT: heat pain tolerance threshold. CI: confidence interval. PPT: pressure pain threshold. PP5: pressure at VAS = 50/100. Ln: natural logarithm. N: Newton. HPT was square-root transformed, while HPTT and PP5 were power transformed, and PPT was log transformed, to improve normality of residuals. Coefficients presented are transformed and should be interpreted as such. The constant refers to mean thresholds for the control group for the habitual sleep condition. Simple effect of group: change in thresholds in migraineurs compared with controls for the habitual sleep condition. Simple effect of sleep: change in threshold after sleep restriction (SR) compared to habitual sleep for controls. The interaction refers to the difference between the effect of SR compared to habitual sleep in migraine compared to controls. Random effects are presented as variances. Random parameters and covariance matrices were included based on likelihood ratio (LR) tests. We used maximum likelihood estimation (MLE) and an unstructured variance-covariance matrix for HPT, whereas restricted maximum-likelihood estimation (REML) was used for HPTT, PPT, and PP5. We used the sandwich estimator for HPT, to account for less than normally distributed residuals. [a]$p = 0.071$. [b]$p = 0.061$.

Supplementary Table S6. Model specifications for the warm detection threshold (WDT, secondary response) in the primary and secondary analyses, using 24- and 48-hour cutoffs for the preictal phase, respectively.

WDT (◦C$^{-0.2}$)	Primary analysis		Secondary analysis	
	Coef.	[95 % CI]	Coef.	[95 % CI]
Simple effects				
Group	-0.025	[-0.063, 0.013]	-0.026	[-0.064, 0.011]
Sleep	0.007	[-0.028, 0.013]	-0.007	[-0.027, 0.013]
Interaction				
Group x Sleep	0.002	[-0.028, 0.033]	0.013	[-0.016, 0.042]
Constant	0.928	[-0.904, 0.953]	0.928	[0.903, 0.953]
Random effects				
Level 2: subject (intercept)		0.005	0.005	
Level 2: session (slope)		0.002	0.001	
Level 1: residuals		0.003	0.003	

CI: confidence interval. WDT was power transformed to improve normality of residuals. Coefficients presented are transformed and should be interpreted as such. The constant refers to mean thresholds for the control group for the habitual sleep condition. Simple effect of group: change in thresholds in migraineurs compared with controls for the habitual sleep condition. Simple effect of sleep: change in threshold after SR compared to habitual sleep for controls. The interaction refers to the difference between the effect of SR compared to habitual sleep in migraine compared to controls. Random effects are presented as variances. Random parameters and covariance matrices were included based on likelihood ratio (LR) tests. We used maximum likelihood estimation (MLE) and the sandwich estimator for WDT.

Supplementary Table S7: Model specifications for explorative models including clinical sleep variables, and comparison between non-sleep-related (NSM) and sleep-related migraine (SM).

	Clinical variable			Random effects			
	Main effects	Interaction	Constant	Level 2: Subject (intercept)	Level 2: Session (slope)	Level 1: Residuals	Covariance: Subject, session
Clinical variable	Coefficient [95 % CI]	Coefficient [95 % CI]	Coefficient [95 % CI]	Estimate	Estimate	Estimate	Estimate
KSS[a]							
HPT ($^\circ C^{0.5}$)	-0.001 [-0.032, 0.031]		2.648 [2.421, 2.874]	0.260	0.155	0.035	-0.101
PPT (lnN)	-0.012 [-0.029, 0.006]		3.151 [3.000, 3.307]	0.157		0.045	
HPTT ($^\circ C^2$)	-1.409 [-4.746, 1.929]		283.5 [255.7, 311.4]	4277.1	1318.6	585.7	
ESS[b]							
HPT ($^\circ C^{0.5}$)	-0.013 [-0.038, 0.012]		2.702 [2.421, 2.984]	0.249	0.163	0.034	-0.102
PPT (lnN)	0.013 [-0.013, 0.038]		3.019 [2.788, 0.025]	0.159		0.044	
HPTT ($^\circ C^2$)	1.235 [-3.136, 5.607]		268.3 [226.4, 310.2]	4155.7	1369.3	604.0	
ISS[c]							
HPT ($^\circ C^{0.5}$)	0.018 [-0.031, 0.067]		2.582 [2.332, 2.840]	0.257	0.163	0.034	-0.103
PPT (lnN)	-0.017 [-0.060, 0.025]		3.190 [2.974, 3.407]	0.161		0.045	
HPTT ($^\circ C^2$)	1.000 [-6.677, 8.677]		276.8 [239.9, 313.7]	4332.7	1331.9	596.2	
NSM vs. SM							
HPT ($^\circ C^{0.5}$)	0.220 [-0.126, 0.567]	-0.329 [-0.808, 0.149]	2.593 [2.387, 2.799]	0.221	0.190	0.032	-0.091
PPT (lnN)	-0.019 [-0.308, 0.271]	-0.012 [-0.231, 0.207]	3.221 [3.070, 3.373]	0.148		0.036	
HPTT ($^\circ C^2$)	21.62 [-24.42, 67.66]	-11.59 [-61.69, 38.51]	240.1 [212.2, 268.1]	4067.9	2049.5	591.0	-657.3
	Main effects	Interaction[d]	Constant	Level 2: Subject (intercept)	Level 2: TST (slope)	Level 1: Residuals	Covariance: Subject, TST
TST[e]		Group x TST					
HPT	0.001 [-0.000, 0.002]	-0.001 [-0.001, 0.003]	2.612 [2.441, 2.783]	0.926	0.000	0.037	-0.002
PPT	-0.000 [-0.000, 0.000]	0.000 [-0.000, 0.001]	3.142 [2.996, 3.288]	0.159		0.045	
HPTT	0.032 [-0.042, 0.106]	0.021 [-0.129, 0.172]	277.6 [252.3, 302.9]	10225	0.045	627.1	-16.28

CI: confidence interval. [a]KSS: Karolinska sleepiness scale: score 1-9. HPT: heat pain threshold. PPT: pressure pain threshold. Ln: Natural logarithm. N: newton. HPTT: heat pain tolerance threshold. [b]ESS: Epworth sleepiness scale (ESS, score 0-3 for eight questions, summed to a total of 24) [c]Insomnia symptom score (ISS, score 0-3 for four questions regarding insomnia symptoms, summed to a total of 12. [e]TST: Total sleep time. Model specifications for exploratory analyses, were we extended the models on HPT, PPT and HPTT. To improve normality of residuals, HPT was square-root transformed, PPT was log transformed, and HPTT was power transformed. Coefficients presented are transformed and should be interpreted as such. The constant refers to mean thresholds for the control group for the habitual sleep condition. Main effect of clinical variable: change in thresholds due to clinical variable. The interaction refers to the combined effect of the clinical variable and sleep restriction on thresholds. For KSS, ESS, and ISS, no interaction term was included. Main effects of group and sleep condition were included in the models, but are omitted here, as the main effect of the clinical variables was the focus of these analyses. Hence, significant effects represent an effect of the clinical variable on thresholds. For the TST analysis, the interaction between TST and group (migraine vs. control) was also included. Random effects are presented as variances. Random parameters and covariance matrices were included based on likelihood ratio (LR) tests. An unstructured variance-covariance matrix was used for all cases of HPT. We used maximum likelihood estimation (MLE) for HPT and HPTT except for 'NSM vs. SM', whereas restricted maximum-likelihood estimation (REML) was used for PPT and HPT and HPTT in 'NSM vs. SM'. For HPT and HPTT we used the sandwich estimator in all models, to account for less than normally distributed residuals, except for 'NSM vs. SM'. The Kenward-Roger approximation for small sample inference was used in the 'NSM vs- SM'-analysis. TST was recorded using actigraphy. Rest intervals defined by the actigraphy software were corrected semi-manually in a hierarchal manor, using the rest intervals, actigraphy event marker for 'lights off' and 'lights on', 'lights off' and 'lights on' from sleep diary, and light and activity levels from actigraph. [d]Interaction for TST: The interaction refers to the combined effect of migraine and TST on thresholds.

Supplementary Table S8: Model specifications for explorative models for selected migraine specific clinical variables for HPT, PPT, and HPTT.

	Main effects	Interaction	Constant	Random effects			
	Clinical variable	Clinical variable x sleep		Level 2: subject (intercept)	Level 2: session (slope)	Level 1: residuals	Covariance: (subject, session)
Clinical variable	Coefficient [95 % CI]	Coefficient [95 % CI]	Coefficient [95 % CI]	Estimate	Estimate	Estimate	Estimate
Duration of migraine attack[a]							
HPT (°C$^{0.5}$)	0.002 [-0.011, 0.015]	-0.003 [-0.023, 0.017]	2.680 [2.491, 2.868]	0.236	0.171	0.032	-0.094
PPT (lnN)	0.002 [-0.007, 0.010]	0.004[1] [-0.001, 0.009]	3.216 [3.086, 3.347]	0.144		0.035	
HPTT (°C^{2})	1.303[2] [0.008, 2.598]	-0.323 [-2.441, 1.795]	247.7 [224.7, 270.7]	3624.0	1824.8	594.2	
Years with diagnosis[b]							
HPT (°C$^{0.5}$)	0.000 [-0.015, 0.016]	0.006 [-0.017, 0.029]	2.543 [1.795, 3.290]	0.226	0.174	0.032	-0.090
PPT (lnN)	0.007 [-0.009, 0.024]	0.003 [-0.004, 0.011]	3.402 [2.683, 4.121]	0.148		0.036	
HPTT (°C^{2})	0.875 [-1.873, 3.622]	2.276[3] [-0.090, 4.643]	244.1 [220.7, 267.4]	3767.6	1507.8	594.5	
Hours to next attack							
HPT (°C$^{0.5}$)	-0.000 [-0.003, 0.003]	0.001 [-0.003, 0.004]	246.2 [2.491, 2.869]	0.232	0.178	0.032	-0.096
PPT (lnN)	0.000 [-0.001, 0.001]	-0.001 [-0.004, 0.001]	3.212 [3.080, 3.344]	0.145		0.036	
HPTT (°C^{2})	-0.078 [-0.471, 0.315]	-0.245 [-0.668, 0.178]	246.2 [220.7, 272.3]	3972.1	1834.2	593.9	
Headache intensity during attacks[c]							
HPT (°C$^{0.5}$)	0.328[4] [0.042, 0.614]	0.405[5] [0.009, 0.801]	2.578 [2.229, 2.926]	0.231	0.171	0.032	-0.118
PPT (lnN)	0.097 [-0.200, 0.395]	0.072 [-0.127 0.272]	3.171 [2.899, 3.444]	0.145		0.036	
HPTT (°C^{2})	40.18[6] [-1.409, 81.78]	3.883 [-45.97, 53.73]	217.2 [178.3, 256.1]	3633.8	1981.2	594.5	
Frequency of attacks[d]							
HPT (°C$^{0.5}$)	-0.013 [-0.334, 0.307]	-0.387 [-0.869, 0.094]	2.618 [2.435, 2.800]	0.228	0.154	0.032	-0.090
PPT (lnN)	-0.014 [-0.294, -0.265]	0.009 [-0.166, 0.184]	3.223 [3.067, 3.380]	0.148		0.036	
HPTT (°C^{2})	-3.788 [-47.29, 39.71]	-40.11 [-89.70, 9.490]	241.1 [214.5, 267.8]	3939.9	1610.3	595.1	
Intensity of photophobia[c]							
HPT (°C$^{0.5}$)	-0.255 [-0.593, 0.083]	0.378 [-0.080, 0.832]	2.973 [2.605, 3.341]	0.183	0.127	0.032	
PPT (lnN)	-0.018 [-0.287, 0.251]	-0.161[7] [-0.296, 0.027]	3.169 [2.948, 3.389]	0.143		0.033	
HPTT (°C^{2})	1.561 [-45.36, 48.48]	17.07 [-38.95, 73.09]	251.5 [203.4, 299.7]	3898.4	1933.8	593.9	
Frequency of photophobia[e]							
HPT (°C$^{0.5}$)	-0.144 [-0.471, 0.183]	0.006 [-0.623, 0.635]	2.779 [2.346, 3.211]	0.230	0.176	-0.096	0.032
PPT (lnN)	-0.014 [-0.289, 0.261]	0.133 [-0.062, 0.327]	3.281 [3.035, 3.526]	0.15		0.035	
HPTT (°C^{2})	27.64 [-17.63, 72.90]	6.929 [-70.57, 84.43]	229.7 [172.4, 287.1]	3757.0	1980.7	594.4	
Intensity of phonophobia[c]							
HPT (°C$^{0.5}$)	-0.064 [-0.367, 0.240]	0.147 [-0.239, 0.533]	2.724 [2.397, 3.052]	0.211	0.174	0.032	-0.082
PPT (lnN)	-0.075 [-0.337, 0.187]	0.002 [-0.157, 0.161]	3.246 [3.055, 3.437]	0.152		0.035	
HPTT (°C^{2})	-5.040 [-50.59, 40.51]	3.037 [-40.72, 46.80]	247.4 [212.6, 282.2]	3971.4	1975.2	597.2	
Frequency of phonophobia[e]							
HPT (°C$^{0.5}$)	-0.053 [-0.363, 0.256]	-0.036 [-0.396, 0.468]	2.717 [2.354, 3.080]	0.231	0.179	0.032	-0.096
PPT (lnN)	.0.030 [-0.282, 0.221]	0.072 [-0.094, 0.238]	3.261 [3.063, 3.459]	0.147		0.035	
HPTT (°C^{2})	-6.308 [-50.04, 37.42]	-4.751 [-53.54, 44.04]	248.3 [210.2, 286.4]	3911.7	1967.9	594.3	

CI: confidence interval. HPT: heat pain threshold. PPT: pressure pain threshold. Ln: natural logarithm. N: Newton. HPTT: heat pain tolerance threshold. Model specifications for exploratory analyses, were we extended the models on HPT, PPT, HPTT. To improve normality of residuals, HPT was square-root transformed, PPT was log transformed, and HPTT was power transformed. Coefficients presented are transformed and should be interpreted as such. The constant refers to mean thresholds for the control group for the habitual sleep condition. Main effect of clinical variable: change in thresholds between the groups for both sleep conditions. The interaction refers to the combined effect of the clinical variable and sleep restriction (SR) on thresholds. Main effects of sleep condition were included in the models, but are omitted here, as the main effects (and simple effects in case of significant main effects or interactions) of the clinical variables and the interaction were the foci of these analyses. Hence, significant effects represent an effect of the clinical variable or a different effect of sleep restriction for each of the levels of the clinical variable. Random effects are presented as variances. Random parameters and covariance matrices were included based on likelihood ratio (LR) tests. An unstructured variance-covariance matrix was used for all cases of HPT, except for the 'intensity of photophobia'-model. We used maximum likelihood estimation (MLE) for HPT and HPTT, whereas restricted maximum-likelihood estimation (REML) was used for PPT. For HPT, HPTT, as well as the model including intensity of photophobia during attacks and PPT, we used the sandwich estimator, to account for less than normally distributed residuals. [a]Average duration of headache with or without use of medication. [b]Age was included as a covariate in these analyses but did not produce significant results. [c]1: mild/moderate, 2: severe. [d]1: < 4 days/month 2: > 4 days/month. [e]1: < ¾ of attacks 2: almost always. [1]p = 0.085. [2]p = 0.049. [3]p = 0.059. [4]p = 0.025. [5]p = 0.045. [6]p = 0.058. [7]p = 0.019.

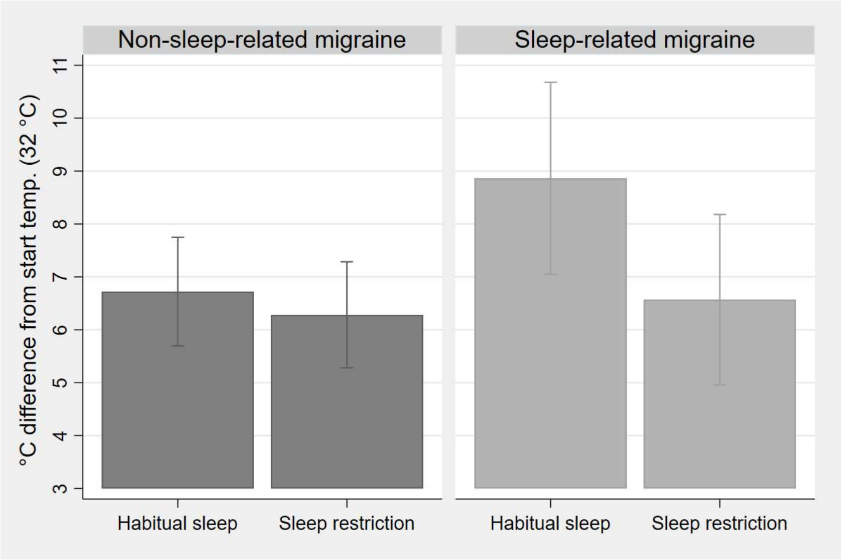

Supplementary Figure S2. Heat pain threshold (HPT). Graphical display of estimated margins with 95 % confidence intervals from a multilevel model, with the effect of habitual vs. restricted sleep (SR) in migraineurs with non-sleep-related migraine (NSM) compared to sleep-related migraine (SM). There was a main effect of reduced HPT after SR (p = 0.040), related to lower HPT after SR in the SM group (p = 0.0595). HPT tended to be lower in the NSM group for the habitual sleep condition (p = 0.071). The SM group was small (see Supplementary Table S9) and the results should be interpreted with caution.

Supplementary Table S9. Number of included test days for HPT, PPT and HPTT in an exploratory analysis comparing the effect of SR in non-sleep-related migraine (NSM) and sleep-related migraine (SM).

			HPT and HPTT				PPT	
	N		Habitual sleep	Sleep restriction	N		Habitual sleep	Sleep restriction
Migraineurs								
NSM	27	n	22	22	29	n	24	23
SM	9	n	6	6	10	n	6	7

HPT: heat pain threshold. HPTT: heat pain threshold. PPT: pressure pain threshold. N = number of interictal test subjects that completed at least one test day; either one after habitual sleep, sleep restriction, or two, i.e. after each sleep condition. n = number of interictal test days after each sleep condition.

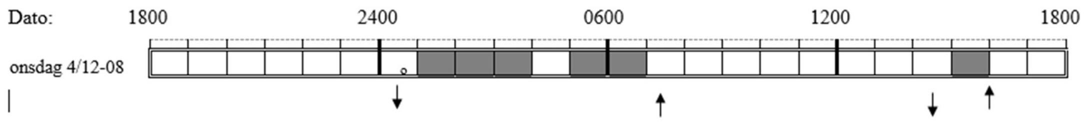

Supplementary Figure S3. Example of one day/night from the sleep diary used in our study. The sleep diary is written in Norwegian (Dato = date; onsdag = Wednesday). The participants were instructed to fill out information about the previous night every morning. The figure below is an example of one day/night from the sleep diary. ↓: Lied down in bed. ↑ Woke up. ₒ: Turned off the lights. Black squares: Sleep. White squares: Awake.

Headache diary

The headache diary was a form with one row for each day and columns for the following information: Date, headache start and stop, headache intensity, type of aura if any, nausea, vomiting, phonophobia, photophobia and use of medications. The participants could also indicate if they recognised the headache attack as a migraine attack, and if the headache attack resulted in sick leave.

Data analysis and statistical analysis

Clinical subgroups

Based on questionnaires, migraineurs were divided into clinical subgroups; intensity of headache as mild, moderate, severe, or extreme; photophobia and phonophobia during attacks as; not present, mild, moderate or severe; frequency of attacks as 1-3, 4-7, 8-14, or > 15 days/month; frequency of photophobia and phonophobia during attacks as < ¼, < ½, or ¾ of attacks, or almost always; as SM if headaches usually started "upon waking" or "during the night (waking me up)", and as NSM if headaches usually started "during daytime before noon", "during daytime after noon", or "no regular onset time" [17]. Due to small groups, relevant clinical variables were aggregated into two groups; intensity of headache, photophobia and phonophobia during attacks as mild/moderate or severe; frequency of attacks as < or > 4 days/month; frequency of photophobia and phonophobia during attacks as ≤ ¾ of attacks or almost always. Because the SM group was small (n = 9, supplementary Table S9) we used the Kenward-Roger correction for small sample inference in the NSM vs. SM-analysis.

Correcting the rest interval in actigraphy

Total sleep time was collected using actigraphy. The rest intervals defined by the actigraphy software were corrected semi-manually in a hierarchal manor, using the rest intervals, actigraphy event marker for 'lights off' and 'lights on', 'lights off' and 'lights on' from sleep diary, and light and activity levels from actigraph.

Model specifications

We used STATA version 15.1 (StataCorp LLC) and multilevel models in this study. Multilevel models are often used in data with hierarchal structures, where data are nested in levels or

clusters (i.e. students in classes in schools, or in our case repeated measurements in subjects). Observations in students in the same class (level, cluster) cannot be assumed to be independent, and multilevel models accounts for this by considering some coefficients as random in addition to traditional fixed coefficients [23]. These can be random intercepts and random slopes for groups, i.e. allowing intercepts and slopes to be different for different groups within levels. Hence, multilevel models are also termed hierarchal models, mixed models, random-effects models, random-coefficient models, and mixed effects models [23]. One advantage of multilevel models is their handling of missing values.

Fixed effects were decided a priori on the basis of hypotheses, while random parameters and covariance matrices were included based on likelihood ratio (LR) tests [23]. We categorised the analyses in a primary, a secondary, and an exploratory analysis: Using a 24-hour cutoff for the preictal phase in the primary analysis [11, 12], a 48-hour cutoff in the secondary analysis [14], and an exploratory analysis investigating addition of clinical migraine and sleep parameters in models from the primary analysis. Marginal means with 95 % confidence intervals and p-values from the multilevel models are shown in Table 4 and 5 for the primary analysis, and in Supplementary Table S2 and S3 for the secondary analysis.

We used exponential transformation on WDT, HPTT, and PP5, square-root transformation on HPT, and logarithmic transformation on PPT. The transformation yielding the best fit for each of the dependent variables was decided based on the Box-Cox transformation.

In selected cases in the primary, secondary, and exploratory analyses we used the sandwich estimator to estimate robust variances. This was done to account for cases with less than normally distributed residuals [23]. We aimed to use restricted maximum-likelihood estimation (REML) in all analyses, as REML is less biased for balanced data compared to maximum-likelihood estimation (MLE) [23]. However, REML is incompatible with the sandwich estimator and REML was therefore only used in cases where the sandwich estimator was not used, i.e. in cases with satisfactory normal distribution. Model specifications, random parameters and use of MLE or REML for each of the models in the primary, secondary, and exploratory analyses are shown in Supplementary Table S4-S8, respectively.

In the primary analysis, MLE and the sandwich estimator were used for WDT, HPT, and HPTT, and REML for PPT and PP5. In the secondary analysis, MLE and the sandwich

estimator were used for WDT and HPT, whereas REML was used for HPTT, PPT, and PP5. In the exploratory analyses, MLE and the sandwich estimator was used for HPT and HPTT, except for the intensity of photophobia and non-sleep-related migraine (NSM) vs. sleep-related migraine (SM) analyses, where REML was used. REML was used for PPT except in the intensity of photophobia analysis, where MLE and the sandwich estimator was used.

Due to safety considerations, the upper limit of the HPTT was set at 52 °C. Several of the included recordings reached this limit (8.9 %), and therefore we do to know what the 'true' thresholds would be in the absence of an upper limit. Such ceiling effects can lead to biased parameter estimation, perhaps especially in longitudinal studies. Consequently, we conducted an additional regression analysis on HPTT, using the Tobit regression model, and defining measures ≥ 52 °C as censored. The Tobit regression model assumes a specific distribution of the 'true values' and performs better in the case of censored data in a longitudinal study design [22]. There is some added difficulty in estimation of parameters when using Tobit regression models, that becomes relevant with complex models and/or in the presence of several random coefficients [22]. Our models are quite simple, including few random coefficients, so the use of a Tobit regression model seems appropriate in our case. The usefulness is also dependent on the proportion of censored measurements, where a low proportion will lead to increased similarity between the Tobit regression model and a standard multilevel model [22]. There were no relevant differences between results from the standard multilevel model and the Tobit regression model for HPTT.
